# Extracellular Fibrils of Pathogenic Yeast *Cryptococcus gattii* Are Important for Ecological Niche, Murine Virulence and Human Neutrophil Interactions

**DOI:** 10.1371/journal.pone.0010978

**Published:** 2010-06-07

**Authors:** Deborah J. Springer, Ping Ren, Ramesh Raina, Yimin Dong, Melissa J. Behr, Bruce F. McEwen, Samuel S. Bowser, William A. Samsonoff, Sudha Chaturvedi, Vishnu Chaturvedi

**Affiliations:** 1 Wadsworth Center, New York State Department of Health, Albany, New York, United States of America; 2 Department of Biomedical Sciences, School of Public Health, University at Albany, Albany, New York, United States of America; 3 Biology Department, Syracuse University, Syracuse, New York, United States of America; The Research Institute for Children at Children's Hospital New Orleans, United States of America

## Abstract

*Cryptococcus gattii*, an emerging fungal pathogen of humans and animals, is found on a variety of trees in tropical and temperate regions. The ecological niche and virulence of this yeast remain poorly defined. We used *Arabidopsis thaliana* plants and plant-derived substrates to model *C. gattii* in its natural habitat. Yeast cells readily colonized scratch-wounded plant leaves and formed distinctive extracellular fibrils (40–100 nm diameter ×500–3000 nm length). Extracellular fibrils were observed on live plants and plant-derived substrates by scanning electron microscopy (SEM) and by high voltage- EM (HVEM). Only encapsulated yeast cells formed extracellular fibrils as a capsule-deficient *C. gattii* mutant completely lacked fibrils. Cells deficient in environmental sensing only formed disorganized extracellular fibrils as apparent from experiments with a *C. gattii STE12α* mutant. *C. gattii* cells with extracellular fibrils were more virulent in murine model of pulmonary and systemic cryptococcosis than cells lacking fibrils. *C. gattii* cells with extracellular fibrils were also significantly more resistant to killing by human polymorphonuclear neutrophils (PMN) *in vitro* even though these PMN produced elaborate neutrophil extracellular traps (NETs). These observations suggest that extracellular fibril formation could be a structural adaptation of C. *gattii* for cell-to-cell, cell-to-substrate and/or cell-to- phagocyte communications. Such ecological adaptation of *C. gattii* could play roles in enhanced virulence in mammalian hosts at least initially via inhibition of host PMN– mediated killing.

## Introduction

‘Primary’ pathogenic fungi that cause serious diseases in healthy humans occur in relatively specialized niches in nature. This trait differentiates them from ‘opportunistic’ fungal pathogens that afflict immunodeficient individuals, since ‘opportunistic’ pathogenic fungi are more widely distributed in soil and plant vegetable matter. During the last decade, more than 400 species of ‘opportunistic’ fungi have been recognized as pathogens for humans and animals [1]. The broad framework for understanding relationships between natural occurrence of the fungal pathogens and their human infectivity currently rests on the idea of ‘dual use’ factors. It suggests that human pathogenic fungi have attributes (‘virulence factors’) that allow them to survive in soil in competition with other life forms, to cycle through invertebrate hosts such as amoebae and to renew their ability to infect human cells [2], [3]. Not specifically addressed by this idea is whether growth on plants and vegetable matter plays a role in the virulence of pathogenic fungi. Such a scenario needs systematic investigation considering that many bacteria, especially enteric pathogens, acquire ecological fitness through growth on plants [4]. Indeed, the model plant *Arabidopsis thaliana* has proven to be a tractable host for the study of bacterial pathogenesis, especially in efforts to unravel virulence factors that are shared between plant pathogens and animal pathogens [5], [6].

*C. gattii* is an encapsulated yeast that causes pulmonary and cerebromeningeal cryptococcosis. *C. gattii* is an emerging pathogen that has triggered serious public health concerns due to (i) its appearance in previously unknown geographic areas, (ii) its outbreaks among healthy humans, pets, and wildlife, (iii) the intractable nature of cryptococcal disease, and (iv) the difficulty of diagnosis in clinical laboratories. Reliable estimates of cryptococcosis due to *C. gattii* are currently lacking, but one estimate suggests that one-third to one-tenth of cryptococcosis cases worldwide are caused by *C. gattii* [7]. Diagnostic laboratories routinely do not distinguish *C. gattii* from the closely related pathogen *C. neoformans*, since the reagents required are not readily available. Current information on *C. gattii* comes either from environmental surveys, which are patchy and far between or from retrospective evaluation of *C. neoformans*/*C. gattii* clinical isolates.

*C. gattii* is readily distinguished from closely related pathogen *C. neoformans* by occurrence on trees, rather than the pigeon droppings colonized by the latter. Other diagnostic characteristics of *C. gattii* include the presence of distinctive cigar-shaped yeast morphology in the host cerebrospinal fluid, agglutinating serotypes, creatinine assimilation and smooth, elongate, rod shaped basidiospores of the teleomorphic form *Filobasidiella bacillispora* [8]. Any of these characteristics could serve as the starting point for systematic studies into virulence of *C. gattii*, but other questions have arisen. Why do rural to semi-urban forested areas pose higher risks for *C. gattii* infection? How do trees figure in the infectious cycle? What is the nature of the infectious propagules, and how are they spread in the environment? Why are apparently healthy humans and animals so prone to infection by this pathogen?

To date, *C. gattii* has been associated with decayed hollows, in the trunks and/or branches of over 50 species of angiosperms and gymnosperms. The growing body of sampling data supports the idea that *C. gattii* is long established among the fungal flora of native trees in many parts of the world [7]. Some experimental studies also provide evidence for *C. gattii*–plant associations. Huerfano et al. [9] demonstrated that *C. gattii* can survive on and be recovered from experimentally infected stems of almond seedlings 100 days post-inoculation, demonstrative of this pathogen's ability to colonize and thrive on live plants. Previously, we reported that *C. gattii* will grow profusely on wood and wood extracts from a variety of trees [10]. This fungal growth was heavily melanized, which was significant as melanin is a known virulence factor. A mutation in transcription factor, Ste12α, caused impaired growth and loss of pigmentation on plant-based media, and a concomitant loss of virulence in a murine model of cryptococcosis. These experiments suggested a link between *C. gattii* ecological fitness and virulence. Xue et al. [11] reported that *C. neoformans* and *C. gattii* can colonize, cause leaf chlorosis, and produce basidiospores on young seedlings of *A. thaliana* and *Eucalyptus camaldulensis* in the laboratory; by extensions, fungal infectious propagules are to be produced on plants in nature [11]. Overall, preliminary work suggests that study of *C. gattii*–plant interactions could elucidate unknown facets of *C. gattii* biology and virulence.

We set out to develop an experimental system that captures native habitat and growth of wild-type *C. gattii*; use of enriched culture media to grow laboratory strains could mask the appearance of certain attributes, especially those that appear in nature early in the establishment of infection in the humans and animals. Further experiments focused on interactions between fungal cells and human phagocytes (PMN), to elucidate the factors that determine if the pathogen persists or is eliminated from the host.

## Materials and Methods

### Ethics Statement

Blood collection procedures from human volunteers complied with the New York State Department of Health Institutional Review Board (IRB) guidelines. Informed consent was obtained from the volunteers prior to the collection of specimens. All animal studies were conducted under full compliance with the guidelines of the Wadsworth Center's Institutional Animal Care and Use Committee (IACUC) in facilities accredited by the Association for Assessment and Accreditation of Laboratory Animal Care (AAALAC). The protocol approval numbers were 06-333 and 09-333. A daily animal welfare chart was maintained to monitor any overt signs of pain and illness and all animals were promptly euthanized according to recommended institution procedures.

### Fungal Strains

*C. gattii* NIH444 (ATCC32609; serotype B, *MATα*), the wild-type strain isolated from cerebrospinal fluid (CSF), was a gift from Dr. K.J. Kwon-Chung (National Institutes of Health, Bethesda, MD). We have previously described this strain as optimum for *C. gattii* molecular pathogenesis studies [12]. *C. gattii ste12αΔ*, a mutant strain with targeted knockout of the transcription factor STE12α, was included as it exhibits reduced colonization of woody substrates, and reduced pathogenicity in mice as a result of defective environmental sensing [10]. We also included a capsular mutant of *C. gattii* (*cap59Δ*;), because the capsule of *C. neoformans* has critical roles in fungal biology and virulence [3], [13]. The *C. gattii cap59Δ* mutant was constructed by homologous recombination of the *cap59::NAT* disruption cassette based on sequences from *C. neoformans CAP59* [14]. The PCR amplifications, biolistic transformation and confirmation of acapsular morphology was according to standard methods [10], [15], [16].

### Growth Media

*C. gattii* cells were cultured in YPD broth at 30°C with shaking (180 rpm), and were maintained in YPD agar and preserved at −70°C in 15% sterile glycerol or in liquid N_2_. Un- treated black cherry (*Prunus serotina*) wood chips were obtained from Roger Dziengeleski, Finch, Pruyn & Co., Inc. (Glens Falls, NY). Leaf agar was prepared by mincing of 10 g of *A. thaliana* leaves into small pieces with scissors, and addition of 2% agar with 0.1% glucose followed by autoclaving for 15 min at 121°C [10].

### *Arabidopsis thaliana*

*A. thaliana* ecotype Columbia (Col-0) and Landsberg erecta (Ler-0), and various mutant lines *eds1* (enhanced disease susceptibility 1; lipase/signal transducer/triacylglycerol lipase), *nahG* (transgenic line degrading salicylic acid; SA), *npr1* (nonexpressor of PR genes 1; pathogenesis-related 1), *sid1* (SA-induction deficient), *rpm1* (resistance to *Pseudomonas syringae* pv maculicola 1), were grown in a greenhouse at the Biology Department, Syracuse University, Syracuse, NY [17], [18], [19], [20]. Four- to six-week old plants were transferred to the Mycology Laboratory of the Wadsworth Center where they were maintained at 20–23°C with a 12 hr light/dark cycle under 50–70% humidity, in a modified incubator with HEPA filtration.

### Plant inoculation, harvest and light microscopy

*C. gattii* cells were subcultured twice in YPD broth at 30°C with 180 rpm shaking and were then collected by centrifugation (3 min at 3,800 rpm), washed twice in deionized sterile water (DSW) and re-suspended to a final concentration of 1.0×10^6^ cells/ml, by counting in a hemacytometer. *A. thaliana* plants from the growth chamber were placed in a sealed carrier case and moved to a BSL-2 safety cabinet. Four to six leaves on each *A. thaliana* plant were marked with a glass marker. Leaves were lightly wounded on the adaxial leaf surface with a 2–3 mm shallow scratch (without complete puncture) on either side of the mid-vein with a 27-gauge syringe needle [21], [22], [23]. Two 5-µl drops of 10^6^ *C. gattii* cells/ml were placed at the wound site, and allowed to air dry (5–10 min). Plants were replaced in modified growth incubator maintained at 20–23°C, 12 hr light/dark cycle, and 50–70% humidity. Macro-photographic images of *A. thaliana* plants and leaves were obtained with a consumer digital camera.

Infected *A. thaliana* leaves were chosen at random and cut from the plant 7 days post-inoculation and homogenized with a tissue grinder in 1 ml of DSW, and the homogenate was serially diluted. 100-µL of each dilution was plated onto YPD agar and incubated for 3–5 days at 25°C, and colonies were counted for determination of fungal viability.

For light microscopy, leaves were cut from plants 7 or 14 days post-inoculation, fixed in 2% glutaraldehyde (EM grade) in phosphate-buffered saline (PBS) or in 0.2 M sodium cacodylate buffer, pH 7.4, alcohol dehydrated by graded series (25%, 50%, 75%, 95% 100%), and stored for further processing in 100% ethanol at 4°C [24]. Staining of whole leaves was accomplished by soaking of leaves in an aqueous solution of equal parts of Trypan blue (1 mg/ml), lactic acid, deionized water, and glycerol for 4-min [25]. Leaves were destained in chloral hydrate (2.5 g/ml), slide-mounted, and viewed and imaged with a table top scanner and light microscope.

### Growth on plant-based and artificial substrates

A number of natural and artificial substrates were used to study growth characteristics of *C. gattii*. The natural substrates were intended to recapitulate growth of *C. gattii* on plants in nature, and for comparisons to growth on organic or synthetic substrates. Wild-type and *C. gattii cap59Δ* mutant strains were grown to mid-logarithmic phase in YPD broth. Cells were collected by centrifugation, washed two times in minimal asparagine medium, with 1% glucose, and re-suspended in the same medium to a final concentration of 1×10^6^ cells/ml. Wild-type *C. gattii cap59Δ* mutant cells were treated with sodium azide (2 mM), followed by incubation at 65°C for 30 minutes to render them non-viable; these cells were used in experiments that compared effects of viable and non-viable cells. The plant-based and artificial substrates used were: autoclaved black cherry wood shavings (Finch, Pruyn & Co., Inc., Glens Falls, NY), consumer brown paper bag, Whatman #1 qualitative filter paper with 98% cellulose (Whatman Limited, Kent, England), Thermanox® plastic coverslips surface treated for optimal cell adhesion (Nalge Nunc International, Rochester, NY), and glass coverslip (Ted Pella, Inc., Redding, CA) and dialysis membrane. The substrate was placed at the bottom of 18-mm round wells of a 24-well flat-bottom polystyrene cell culture plate. One milliliter of 1×10^6^ cells in minimal asparagine medium, was added to each well in one row of the 24 wells in duplicate. The culture plates were covered and incubated for 24- or 48-hr at 25°C. The cell suspension was removed, and wells were washed 3 times with 0.05% Tween-80 in Tris-buffered saline (TBS), to remove non-adherent cells. Substrates were fixed with 2% glutaraldehyde in 0.2 M sodium cacodylate buffer, pH 7.4 for 24 hr at 4°C, and dehydrated by exposure to a graded ethanol-water series, and stored in 100% ethanol in plates. Average numbers of attached cells were determined by counting cells in four SEM fields per sample.

### Scanning electron microscopy (SEM)

*A. thaliana* leaves inoculated with *C. gattii* were harvested at indicated intervals, fixed in 2% glutaraldehyde (EM grade) in phosphate-buffered saline (PBS) or 0.2 M sodium cacodylate buffer, pH 7.4, alcohol-dehydrated by graded series (25%, 50%, 75%, 95%, 100%), and stored in 100% ethanol. The dehydrated specimens were critical point dried with liquid CO_2_ in a tousimis Samdri 795 drier (Rockville, MD, USA), gold-sputter-coated, observed and imaged with a LEO 1550 Variable Pressure field emission gun SEM (Carl Zeiss SMT, Peabody, MA), according to procedures followed in our laboratory [24]. A semi-quantitative estimate of *C. gattii* attachment to the natural or artificial substrates was obtained similarly to our quantification of mating in SEM images described previously [10]. Briefly, cells were counted both individually and in clusters. A cell cluster was defined as an aggregation of four or more *C. gattii* cells. Mean average counts were obtained from determining the number of *C. gattii* cells in at least four or more SEM images.

### High voltage electron microscopy (HVEM)

*A. thaliana* leaves that have been scratch-wounded and inoculated with *C. gattii* cells were harvested and fixed in 2% glutaraldehyde (EM grade) in 0.2 M sodium cacodylate buffer, pH 7.4 overnight. Leaves were washed three times with 0.2 M sodium cacodylate buffer and stained with 1% osmium tetroxide (OsO_4_) in 0.1 M sodium cacodylate for 1-hr at 4°C, rinsed two times with DSW, and dehydrated through a graded ethanol series [26]. Samples were then embedded in Spurr low-viscosity embedding medium (Polysciences, Inc.; Warrington, PA, USA), which was allowed to polymerize at 70°C for 48-hr. Blocks with embedded *A. thaliana* leaves were cut and trimmed with a hot razor blade, and mounted on pre-formed TEM pegs. Sections were cut approximately 1 µm thick, on a microtome and were mounted on formvar-coated grids. Grid-mounted sections were then stained with 2% uranyl acetate for 60 min, followed by Reynold's lead stain for 20 min; they were then allowed to air dry, and were stored in an EM grid box. Sections were observed and imaged with the 1.2 MeV AEI EM7 MK II high voltage electron microscope (HVEM) at the Wadsworth Center.

### Effects of inhibitors of cytoskeletal proteins

We wanted to examine the roles of actin, tubulins and other cytoskeletal proteins on *C. gattii* microtubes, since these proteins are important determinats of cell shape [27], [28]. *C. gattii* wild-type and *cap59Δ* mutant strain were grown in YPD broth, 30°C, 180 rpm for 12–16 hr and treated with thiabendazole, cytochalasin B, mebendazol, or latrunculin B (details in Suppl. information).

### Murine model of cryptococcosis

Virulence of *C. gattii* was tested in a murine model of cryptococcosis that mimics either progressive pulmonary cryptococcosis (intranasal inoculation, IN) or rapid onset of cerebromeningeal cryptococcosis (intravenous inoculation, IV) as described previously [10], [29], [30]. *C. gattii* cells were grown with two successive passages on YPD agar and *A. thaliana* leaf-agar at 25°C for 4 days. Four- to six-week old male BALB/c mice were obtained from Charles River Laboratories, Inc. Groups of 8 BALB/c mice were inoculated either via IN or IV route with 30 µl or 100 µl DSW containing 10^5^ cells or 10^4^ cells, respectively. Mice were given food and water *ad libitum*, and were observed daily according to a welfare score sheet that addresses appearance of disease symptoms, general malaise, and any apparent pain and discomfort in the infected animals. After signs of progressive poor health or discomfort were noted, the infected animals were euthanized by CO_2_ inhalation followed by cervical dislocation. Data from five infected mice (IN or IV) were used to determine Kaplan-Meyer survival curve using SAS software (SAS Institute, Inc.; Cary, N.C). An assessment of colonization and multiplication within lungs and brain of infected mice was obtained by using three mice from each group, which were euthanized at day 7 (IV) or day 14 (IN) post infection. None of the animals displayed any signs of overt illness at these two time periods. One-half of lung or brain harvested from infected animals was preserved for histopathological analyses, in Bouin's fixative (brain section) or buffered formalin (lungs). The other halves of target organs were used to calculate relative fungal loads. Tissues were weighed and homogenized in 1 ml DSW, serial dilutions were plated on YPD agar, and incubated at 30°C for determination of CFU. Histopathological examinations were done as described previously [10]. Briefly, after 24 hr of fixation, tissues were sectioned, and brain sections were washed in distilled water for several hours. Processing was done in a vacuum infiltration processor, the Tissue-Tek VIP 5 (Sakura Finetech), starting with 70% ethanol and proceeding through a series of dehydrating alcohols and xylenes into paraffin, for 15 min per station. Tissues were then embedded in paraffin blocks and sectioned at a 7-µm (brain tissues) and 3–4 µm (lung tissues) thickness. Sections were stained with hematoxylin & eosin and mucicarmine (Richard Allen Scientific) and examined by light microscopy.

### *C. gattii*–PMN interactions

We have previously shown that *in vitro* interactions between human PMN and *C. gattii* can be used for a complimentary evaluation of virulence outcome in mouse models, and to assess the likely outcome of initial interactions between *C. gattii* and phagocyte cells [10], [29], [31]. We compared *C. gattii* cells grown on leaf agar to *C. gattii* cells grown on YPD agar; a clinical isolate of *Candida glabrata* was used as control as was *C. gattii cap59Δ* mutant. Blood was obtained from human volunteers per protocol approved by the New York State Department of Health Institutional Review Board. PMN were isolated by Ficol-Paque (Pharmacia LKB Biotechnology) centrifugation [10], [29], [30]. PMN were washed twice and resuspended in RPMI-1640. PMN and yeast cells were incubated at a 10∶1 ratio for 4 hr at 37°C under 5% CO_2_, followed by plating on YPD agar for CFU determination [31]. Percent fungicidal activity was calculated as: 1-(CFU experiment ÷ CFU controls) ×100. Results were expressed as means±standard error (SE) from the values from at least four individual blood donors [10], [29]. SEM analysis of *C. gattii*-PMN interactions were carried using Thermanox® plastic cover slips in 24-well flat-bottom tissue culture plates. PMNs and yeast cells were mixed at a 1∶10 ratio, added to each well, and incubated for 4 hr at 37°C under 5% CO_2_. Following incubation, cells were fixed with 2% glutaraldehyde and processed as described earlier.

## Results

### *C. gattii* proliferates on *A. thaliana* leaves

**Figure 1:**
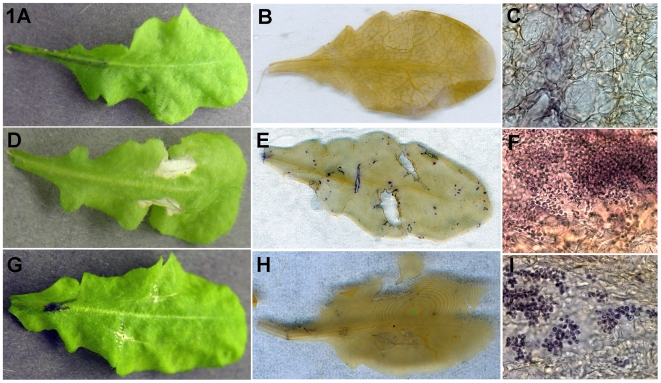
*C. gattii* colonized experimentally inoculated *A. thaliana* leaves. *A. thaliana* leaves on live plants were inoculated with sterile deionized water control (A), *C. gattii* wild type cells (D), or *C. gattii cap59Δ* mutant (G). Gross examination of the control leaves with mock inoculations showed no scars while large chlorotic lesions were seen in leaves inoculated with *C. gattii* wild type cells; much smaller lesions were visible in leaves inoculated with *C. gattii* mutant cells. Further examination of inoculated leaves after fixation and staining showed no significant fungal colonization around inoculation sites in the mock- inoculated and *cap59Δ* mutant-inoculated leaves (B, H), but leaves inoculated with *C. gattii* wild-type cells showed fungal cells around inoculation sites (E). Microscopic examinations of leaves revealed numerous *C. gattii* wild-type cells at the inoculation site and also cells that had dispersed away from the original inoculation (F); in contrast, cap59*Δ* mutant cells were localized at few spots around the inoculation site (I) (100× magnification). Panels A, B and C are from different leaves to illustrate salient features of these observations.

Gross examination of *A. thaliana* ecotype Col-0 leaves inoculated with *C. gattii* wild-type strain showed noticeable chlorotic lesions around the initial wound site. Leaves inoculated with *C. gattii cap59Δ* mutant strain had smaller chlorotic lesions (Figs. 1D, 1G). However, neither of two *C. gattii* strains caused systemic plant disease in contrast to what has been reported for a number of plant pathogenic fungi grown on *A. thaliana* plants [32], [33]. Microscopic examinations of trypan blue-stained leaves for the presence of yeast cells showed *C. gattii* colonization along wound sites, as well as punctuated colonization across the leaf, even distant from the initial inoculum site and wound (Fig. 1E,1F). In contrast, *A. thaliana* leaves inoculated with *C. gattii cap59Δ* mutant had much lower number of yeast cells that were confined to the initial inoculation sites (Figs. 1H and 1I). Control leaves with mock-inoculation did not display any specific staining (Fig. 1B–1C). Yeast cells were stained most heavily at the wound edges in the leaves consistent with observed chlorotic regions (Fig. 1E). Overall, there was ample evidence for *C. gattii* wild-type strain colonization and dispersal from the wound sites on *A. thaliana* Col-0 ecotype leaves. Preliminary examination of another ecotype *A. thaliana* Ler-0 indicated almost two-times more colonization visually as seen on Col-0 ecotype (Fig. S1). Additionally, a number of mutant plant lines derived from *A. thaliana* Col-0 ecotype and deficient in plant defense against fungal pathogens, showed higher colonization of inoculated leaves by *C. gattii* wild-type strain (*eds1*>*nahG*>*sid2*>*npr1*>*rpm1*; Fig. S2).

### *C. gattii* extracellular fibrils on plant leaves

**Figure 2:**
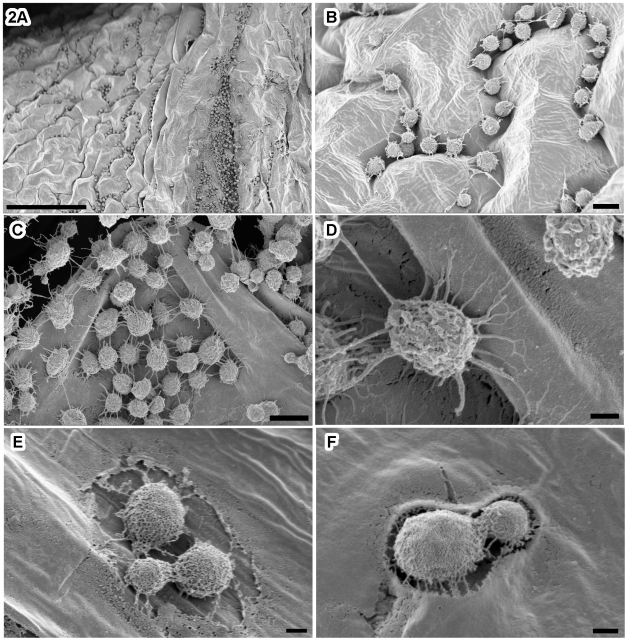
*C. gattii* formed extracellular fibrils on *A. thaliana* leaves. SEM images of infected leaves showed that *C. gattii* colonized wound site and crevices along leaf surfaces (A, B), extracellular fibrils were visible projecting from *C. gattii* cells, connecting yeast cells to each other and to the leaf surface (C, D). Note a yeast cell at higher magnification with prominent extracellular fibrils extending to other yeast cells and to the leaf tissue (D). Higher magnification of *C. gattii*- inoculated leaf surface revealed formation of ‘leaf halo; and ‘pocket’ in *A. thaliana* in some instances (E, F). Scale bar equals 100 µm (A), 5.0 µm (B, C), and 1.0 µm (D, E, F).

Further examinations by SEM revealed that *C. gattii* profusely colonized wounded *A. thaliana* leaves, with the highest numbers of yeast cells present at the wound sites, but significant numbers of cells seen away from the wounds, a pattern consistent with the light microscopic observations (Fig. 2A, B). Although *C. gattii* cells on leaves were intact, and showed buds of various sizes, we could not tell whether colonization away from wound sites resulted by active spread or by contact with other inoculated leaves or possibly by deposits of yeast aerosols in the custom growth chamber. *C. gattii* colonization demonstrated a number of unique features that have not been reported in literature: a) extracellular fibrils of variable lengths on the cells (Fig. 2C, D); b) extensive inter-connected individual yeast cells via extracellular fibrils (Fig. 2B, C), and c) formation of pockets or holes on leaf surfaces around *C. gattii* cells (Fig. 2E, F). The dimensions of the fibrils were measured from SEM images: 40–100 nm length ×500–3000 nm diameter. Further experiments revealed that the incorporation of leaves and other plant based substrate in sterile agar also induced the production of extracellular fibrils similar to those seen on intact leaves (Fig. S3).

**Figure 3:**
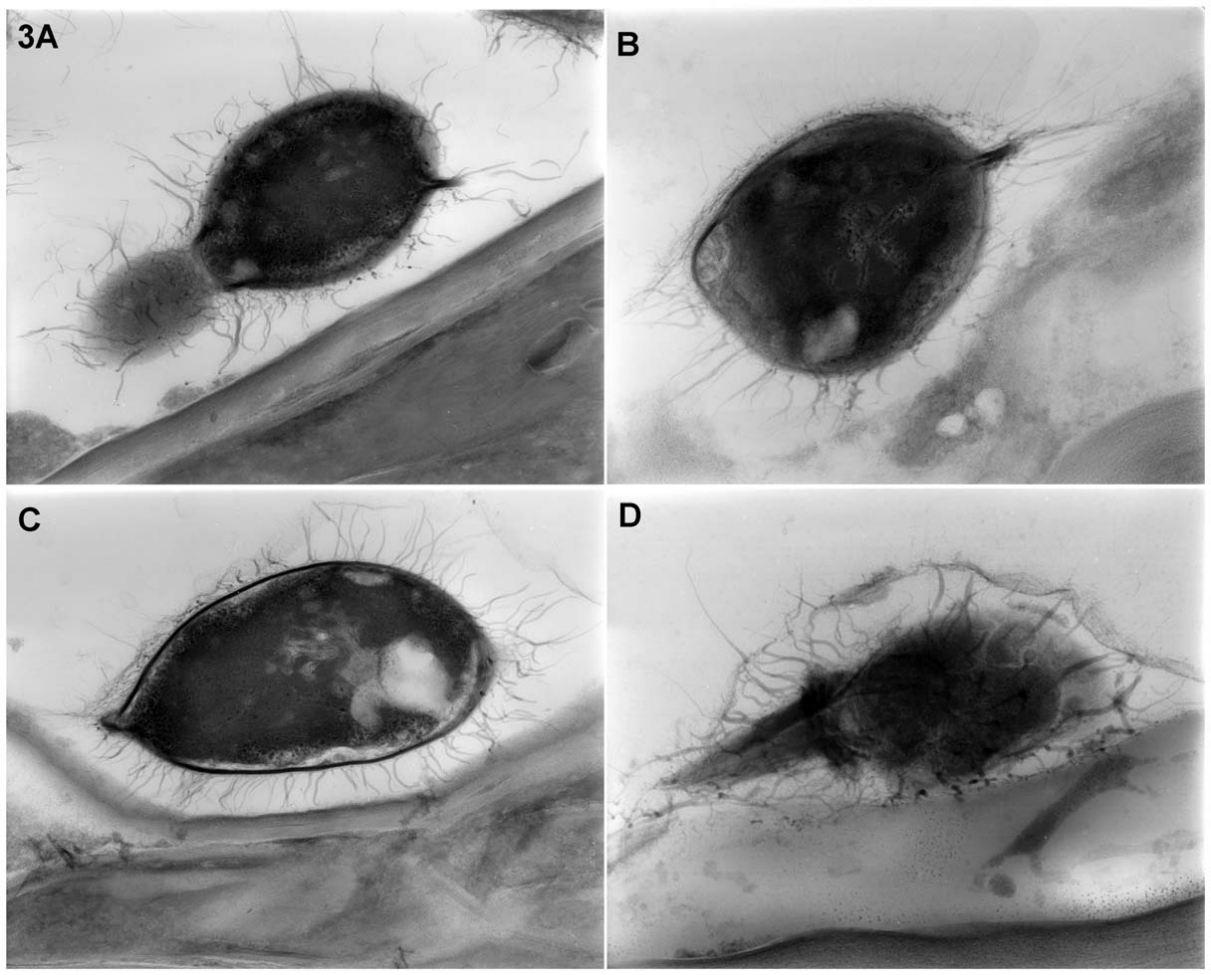
*C. gattii* extracellular fibril organization visualized by high-voltage EM. HVEM examination of infected leaves showed extracellular fibrils covering *C. gattii* cells in close proximity to the leaf tissues (A–D). Both mature and budding *C. gattii* cells produced extracellular fibrils although young buds displayed relatively less projections. The fibrils were uniformly distributed along the entire cell surface.

Further examination of *C. gattii* extracellular fibrils was carried out in HVEM using thick-section microscopy of inoculated leaves (Fig. 3). The sections revealed that extracellular fibrils extended uniformly from cell surfaces in association with leaf tissue (Fig. 3A, D). Both mature and budding cells were covered with fibrils while young buds displayed relatively less projections.

### *C. gattii* mutants have vestigial or no fibrils

**Figure 4:**
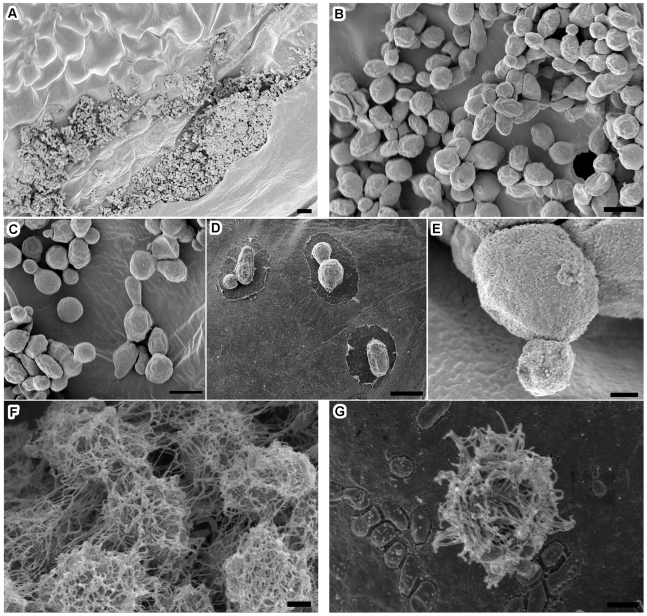
*C. gattii* mutants were defective in extracellular fibril formation. *C. gattii cap59Δ* mutant cells appeared restricted to the initial wound site (A), and no projections were visible on these cells and they appeared clumped together on leaf surface (B, C). Remarkably, pockets and holes in the leaf surfaces were not as prominent in leaves (D). Higher magnification of *C. gattii cap59Δ* mutant cell confirmed the absence of extracellular fibrils (E). *C. gattii ste12αΔ* mutant produced extracellular fibril-like structures, but these were greatly disorganized and did not resemble well-formed features seen in wild-type cells (F, G). Scale bar equal 10 µm (A), 2.0 µm (B, C, D), and 1.0 µm (E, F, G).

Although *C. gattii cap59Δ* mutant showed attachment to the inoculation sites, no extracellular fibrils were seen on these mutant cells (Fig. 4A–E). The growth of *C. gattii cap59Δ* mutant was restricted, and cells appeared in clumps (Fig. 4A, B). The data were consistent with the mutant's reduced production of chlorotic lesions and colonization on *A. thaliana* leaves, as described in the previous section. Other features seen on *A. thaliana* inoculated with *C. gattii* wild-type cells such as pockets and holes in the leaf surfaces were not observed in leaves inoculated with *C. gattii cap59Δ* mutant. The sparse appearance of a digestion-like halo surrounding a few *C. gattii cap59Δ* mutant cells suggested either absence of these structures or their rare occurrence (Fig. 4D).

We also tested *C. gattii ste12αΔ* mutant, to see whether defect in environmental signaling has any role in fungus –plant interactions. As seen in SEM micrographs, the inoculated *C. gattii ste12αΔ* mutant colonized *A. thaliana* leaves and displayed extracellular fibrils (Fig. 4F, G). However, these fibrils were not as well-formed as those seen in *C. gattii* wild-type strain, and colonized leaves did not show pockets or holes.

*C. gattii* cells exposed to cytoskeletal protein-inhibitors namely, thiabendazole, cytochalasin B, mebendazol or latrunculin B demonstrated variable disorganization of extracellular fibrils on live *A. thaliana* leaves (Fig. S4).

### *C. gattii* extracellular fibrils can be induced *in vitro*

**Figure 5:**
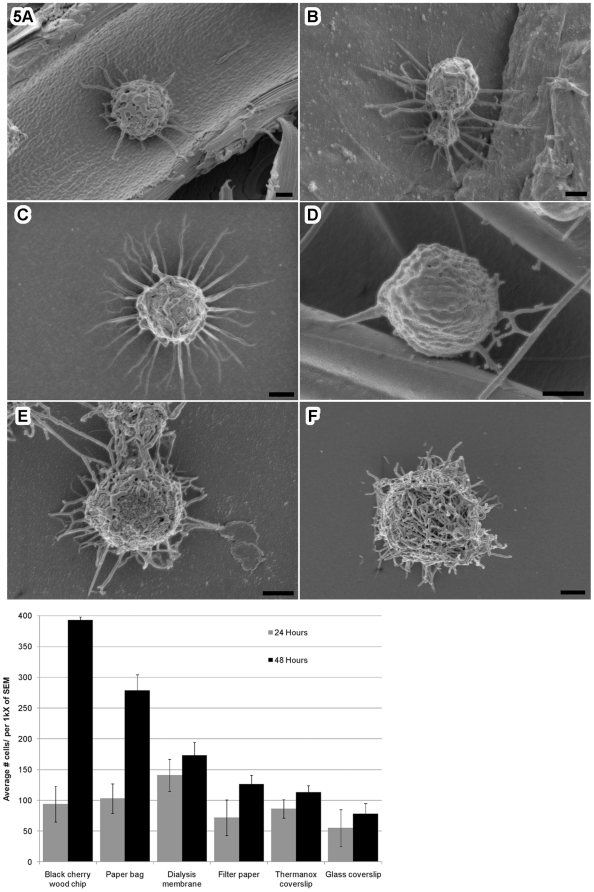
*C. gattii* extracellular fibril formation on plant-based and synthetic substrates. *C. gattii* extracellular fibrils were observed on variety of substrates. SEM images of one representative cell on each substrate is shown after 48 hrs observation period: (A) wood chip from black cherry, (B) paper bag, (C) dialysis membrane, (D) filter paper, (E) Thermanox® coverslip and (F) glass coverslip; scale bar, 1.0 µm. The extent of extracellular fibrils formation on these substrates was determined indirectly by a semi-quantitative attachment assay (details in the methods section). The histogram illustrates that extracellular fibrils were more numerous and well-formed on plant-based substrates and less frequent on artificial substrates (cherry wood chip>brown paper bag>dialysis membrane>filter paper = Thermanox® coverslip>glass coverslip).

*C. gattii* cells were further tested to determine whether extracellular fibrils formation was an exclusive response to a live plant host or these features could also be induced on other substrates. Extracellular fibrils were observed to form on variety of substrates such as cherry wood chips, brown-paper-bag, Thermanox® plastic coverslips, cellulose filter paper, and glass coverslips (Fig. 5A–F). Extracellular fibrils were more numerous and better formed on plant-based substrates than on synthetic substrates and less frequent on artificial substrates. The rates and pattern of colonization of the different substrates between 24–48 hours was as follows: cherry wood chip>brown paper bag>dialysis tubing>filter paper = Thermanox® coverslip>glass coverslip (Fig. 5, histogram). Cytoskeletal protein-inhibitors thiabendazole, cytochalasin B, mebendazol or latrunculin B caused disorganization of extracellular fibrils formation in *C. gattii* cells grown in in vitro assay (Fig. S5).

### *C. gattii* cells with extracellular fibrils are hypervirulent

**Figure 6:**
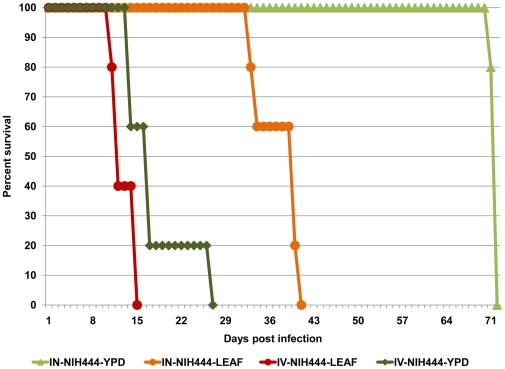
*C. gattii* cells with extracellular fibrils were hypervirulent in murine models of cryptococcosis. *C. gattii* cells grown on YPD agar or leaf agar were used as inoculum in mice models that mimic acute (IV inoculation) or chronic (IN) cryptococcosis and the results were used to plot Kaplan-Meyer survival curves. The survival curves revealed that mice infected with *C. gattii* cells from leaf agar had significantly lower survival (p<0.001) than did mice infected with *C. gattii* cells grown on YPD agar. This hypervirulence trait was even more pronounced when cells grown on leaf agar were used for IN infection (p<0.0001).

Kaplan-Meyer survival curves revealed that mice infected IV with *C. gattii* cells grown on leaf agar had significantly lower survival (*p*<0.001) than did mice infected with *C. gattii* cells grown on traditional YPD agar (Fig. 6). For IN route of infection, the difference in virulence of *C. gattii* cells grown on leaf agar and cells grown on YPD agar was even more significant (*p*<0.0001).

**Figure 7:**
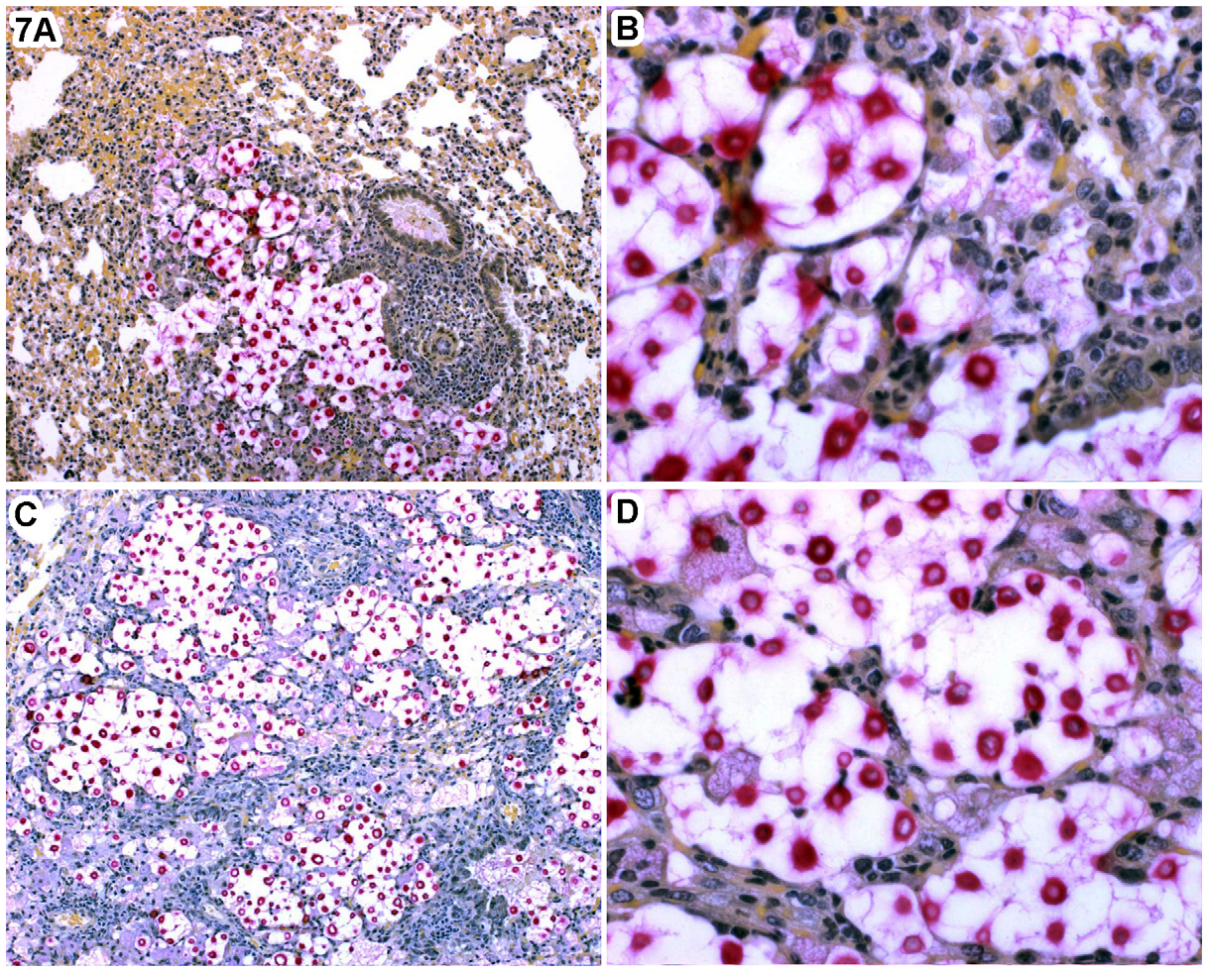
*C. gattii* cells with extracellular fibrils elicited less robust tissue responses. The histopathological results are from the post-mortem examination of lungs of mice infected IN with *C. gattii* cells were taken either from YPD agar (A, B) or leaf agar (C, D). (A) a focus of fungal replication is bordered by intense mixed neutrophil and pulmonary macrophage inflammatory response in alveoli and perivascular connective tissues, Mucicarmine, 100X; (B) Higher magnification showing intense neutrophilic inflammation borders mucicarmine positive *C. gattii* cells, Mucicarmine, 400X; (C) Severe diffuse lung invasion by *C. gattii* from leaf agar in the mouse model of intranasal inoculation. All alveoli are either colonized by the fungus or otherwise compromised by a predominantly suppurative inflammatory response. Mucicarmine, 100X; and (D) Higher magnification of showing numerous mucicarmine positive *C. gattii* cells fill alveoli; there is a weaker neutrophilic response than see in (B). Notably, there are many extracellular organisms with no inflammation, the hallmark of cryptococcosis caused by hypervirulent *C. gattii* strains.

Lungs and brains of mice infected with *C. gattii* cells grown on leaf agar had higher total fungal counts than lungs and brains of mice infected with *C. gattii* cells grown on YPD agar (Fig. S6). This raised the possibility that fungal cells with extracellular fibrils proliferated more rapidly in the infected tissues. Post-mortem examination of mice IN-infected with *C. gattii* cells from leaf agar revealed extensive tissue damage in form of enlarged, mottled lung tissue consistent with extensive pneumonia, while lung and brains of mice infected with YPD grown *C. gattii* cells appeared less damaged (Fig. 7). Notably, more PMNs infiltrated the lungs of mice infected with *C. gattii* cells grown on YPD agar (Fig. 7B) than was observed for PMN infiltration in lungs of mice infected with *C. gattii* cells grown on leaf agar (Fig. 7D). *C. gattii* cells from leaf agar were also numerous in extracellular spaces without the presence of any neutrophils. This raised a possibility that lack of or inhibition of PMN infiltration was related to more tissue damage in mice infected with *C. gattii* cells grown on leaf agar. A comparison of brain sections of IV-infected animals revealed larger numbers and more prominent brain lesions in mice infected with *C. gattii* cells with extracellular fibrils; these lesions also contained relatively more yeast cells than did lesions seen in mice infected with *C. gattii* cells grown on YPD agar (details not shown). This observation was also consistent with more proliferation of *C. gattii* cells with extracellular fibrils. Unfortunately, it was not practicable to examine these infected tissues by electron microscopy. Therefore, we are unable to provide any evidence of the presence or absence of the *C. gattii* extracellular fibrils in the infected murine tissues.

### *C. gattii* with extracellular fibrils are more resistant to PMN killing

**Figure 8:**
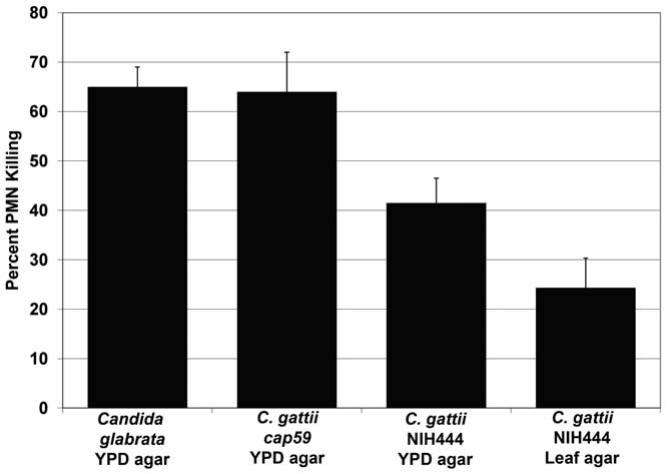
*C. gattii* with extracellular fibrils were more resistant to human PMN killing. *C. gattii* cells grown on leaf agar showed reduced susceptibility to in vitro killing by human PMN in phagocyte interaction assay. The data showed that only 24% of initial 10^3^ yeast cells used in the inoculum were susceptible to PMN-induced killing. In contrast, higher PMN fungicidal activity was seen with *C. gattii cells* grown on YPD agar (41% killing). The two control used in these experiments showed expected high susceptibility to PMN-mediated fungicidal activity: *C. gattii cap59Δ*mutant (64% killing), and *Candida glabrata* (65% killing). Results are mean±SE of at least four independent experiments.

*C. gattii* cells grown on leaf agar were significantly more resistant to *in vitro* killing by human PMN than were *C. gattii* cells grown on YPD agar (Fig. 8; *p*<0.05). Controls cells in this assay including *Candida glabrata* and *C. gattii cap59Δ* mutant were highly susceptibility to PMN killings as expected. PMN assay results were consistent with earlier findings in this study from mouse survival experiments, and these findings raised the possibility that at least one mechanism behind the increased virulence of *C. gattii* cells with extracellular fibrils is their greater ability to escape from PMN-mediated host defenses.

**Figure 9:**
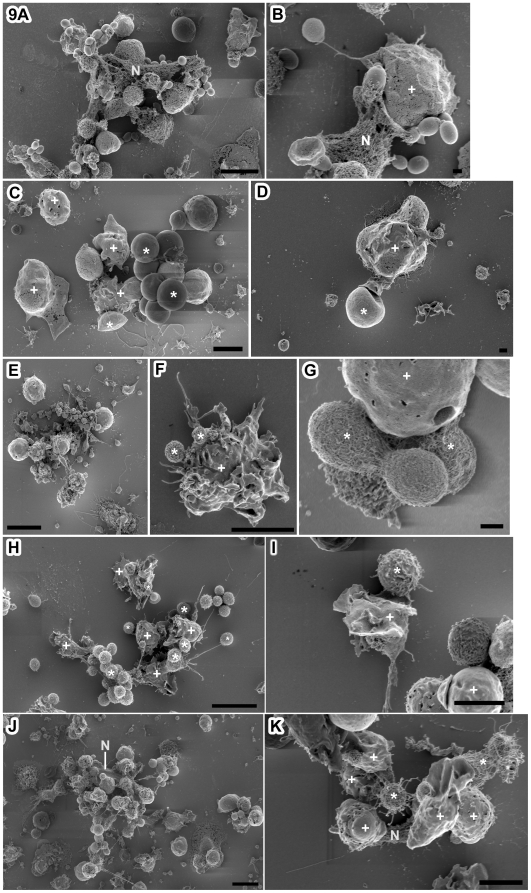
*C. gattii* fibrils interfered with entrapment by neutrophil extra-cellular traps (NETs). SEM analysis revealed that activated PMN made profuse NETs against control organism *Candida glabrata* (A, scale bar 10 µm; B, scale bar 5 µm). Rudimentary to no NETs were visible against *C. gattii cap59*Δ mutant cells (C, scale bar 10 µm; D, 5 µm). *C. gattii* cells grown either in YPD broth (E, F) or on YPD agar (G, H) had no or rudimentary fibrils, and group of 1–5 yeasts were seen around an activated neutrophil, which produced only rudimentary NETs. *C. gattii* grown on leaf agar made extensive fibrils, which extended between yeast cells, and yeast cells and multiple activated PMN (I and K scale bar 10 µm; J, scale bar 5 µm). Symbols: (*) fungal cells, (+ or N) PMN.

By SEM, we further examined roles of *C. gattii* extracellular fibrils in PMN interactions. Neutrophils extracellular traps (NETs) were prominent against control organism *Candida glabrata*. NETs from one neutrophil entrapped many *Candida glabrata* cells (Fig. 9A). This observation was similar to an earlier report on involvement of NETs in PMN- *Candida albicans* interactions [34]. *C. gattii cap59Δ* mutant–neutrophils interactions showed many yeast cells trapped by single neutrophils with NETs (Fig. 9C, D). C. gattii cells grown in standard YPD broth did not form fibrils, and multiple yeast-cells were seen in interactions with one activated neutrophil (Fig. 9E–G). Interaction of *C. gattii* cells grown on YPD agar showed 1–5 yeast cells with an activated neutrophil (Fig. 9H, I). The PMN in these interactions had rudimentary NETs while *C. gattii* cells from YPD agar formed a few extracellular fibrils. Leaf agar grown *C. gattii* cells made extensive extracellular fibrils; these fibrils made yeast cell- yeast cell connections and also connections to many activated PMN (Fig. 9J, K). Notably, *C. gattii* extracellular fibrils appeared to lie atop or extend over PMN.

## Discussion

The present study revealed several unreported aspects of *C. gattii* that have implications for its ecology and virulence. *C. gattii* readily colonized scratch-wounded *A. thaliana* leaves on live plants forming organized layers of fungal cells with unique extracellular fibrils emanating from and between them. Extracellular fibrils were also formed when *C. gattii* cells were grown on plant-based substrates, and on materials promoting cell adhesion. Formation of *C. gattii* extracellular fibrils was associated with hypervirulence in mice infected via IN route, and with an increased resistance to killing by human PMN in vitro. Paradoxically, human PMN NETs were observed in the presence of extracellular fibrils–bearing *C. gattii* cells, but the fibrils apparently conferred enhanced fungal resistance to PMN killing. These observations suggest that *C. gattii* extracellular fibrils formation could be a structural adaptation for cell-to-cell, cell-to-substrate and cell-to- host communication. Pathogenic fungi might possess features enabling them to adapt to their ecological niche; such features could also serve to enhance fungal virulence in the mammalian hosts at least initially via inhibition of host PMN- mediated killing.

Discernment and characterization of extracellular fibrils in *C. gattii* prompted us to search for precedents for such structures, in the literature. Bacteria, of course, have pili and flagella for locomotion and virulence [35]. Other extra-cellular extensions have been extensively recorded from both prokaryotic and eukaryotic cells. In eubacteria, they are variously termed adhesion threads, extracellular appendages, fibrils, and filaments [36], [37], [38], [39] while in mammalian cells they include blebs, filopodia, lamellipodia, podosomes, and nanotubes [40], [41], [42], [43], [44]. *Cryptococcus gattii* extracellular fibrils did not fully share features such as their induction and size with previously recorded descriptions. Among fungi, we first focused on a remarkable study by Varon and Choder [45] who described ‘intercellular connecting fibrils found in starved *Saccharomyces cerevisiae* colonies’. *Saccharomyces cerevisiae* fibrils showed some resemblance to *C. gattii* extracellular fibrils; single fibrils were seen between adjacent cells and they measured 180±50 nm in diameter, while *C. gattii* extracellular fibrils were far numerous, and had diameters of 40–100 nm and 500–3000 nm length. A closer similarity to the extracellular fibrils was suggested by work of Kock and colleagues on oxylipins or oxygenated fatty acids of closely related pathogen *C. neoformans*. Oxylipins are secreted from cells via tubular protuberances emanating from the capsule [46], [47], [48]. Although the protuberances described by this research group appear to be sparse, not as well-formed or preserved, the size measurements in one transmission electron micrograph is in close approximation of the sizes of *C. gattii* fibrils seen in our study [46]. Further studies are needed to confirm the extent and nature of extracellular fibrils in *C. neoformans*.

*Cryptococcus* capsule architecture is currently the topic of intensive study. That the capsule includes apical extensions or distal re-arrangements has been investigated by Casadevall and colleagues, who employed a number of sophisticated methods to demonstrate a loosely organized fibrous material spatially continuous with the capsular polysaccharide of *C. neoformans* [49], [50], [51], [52]. Therefore, we constructed a capsule-deficient strain of *C. gattii*, using a homolog of the *C. neoformans CAP59* gene, for directed disruption, because this gene was originally identified as critical for capsule synthesis [14]. *C. gattii cap59Δ* mutant strain lacked extracellular fibrils and did not colonize *A. thaliana* leaves, or attached to Thermanox® coverslips as efficiently as the wild-type strain. These observations suggested that formation and organization of extracellular fibrils are linked to the intact polysaccharide capsule either directly or indirectly, perhaps via a capsular role in extracellular transport, as has been studied in *C. neoformans* [53]. A series of studies reported by Doering and colleagues demonstrated that intact cell wall α -1–3- glucan is important for the formation of capsule and fibers in *C. neoformans* [54], [55]. Preliminary experiments in our laboratory indicated that formation of extracellular fibrils needed other cellular components. For example, fixation of *C. gattii* cells in paraformaldehyde, but not in glutaraldehyde, caused disruption of capsules and intact extracellular fibrils. Because protein cross-linking is less efficient in paraformaldehyde than in glutaraldehyde, we further investigated this observation by using cytoskeleton protein inhibitors. Varying degrees of extracellular fibril disorganization resulted from these treatments, similarly to what has been reported for a number of eukaryotic appendages and fibrils formed by actin polymerization and tubulin repolymerization [41], [56]. Interestingly, extracellular fibril formation was abnormal in *C. gattii ste12αΔ* mutant strain; STE12 is a fungus specific transcription factor with direct roles in sexual development and environmental sensing [10]. We interpreted these observations to indicate that appropriate environmental clues are critical, if *C. gattii* cells are to commit to developmental pathway (s) leading to extracellular fibrils formation.

We preferred our *A. thaliana* scratch wound model over other inoculation methods, because it is more consistent with natural wounding of plant tissues as a result of herbivory, insect attack, animal damage or abiotic damaging events [57]. Extensive experimental evidence for environmental specialization is available for *C. neoformans*. For example, in landmark studies, Staib and colleagues established that pigeon dropping could provide adequate nutrition for *C. neoformans* asexual and sexual growth, notable because these droppings are a relatively nutrient-poor source for microbial growth [58]. More recently, it was shown that *C. neoformans* undergoes filamentation and sexual mating on pigeon manure agar [59]. Our present studies raise the possibility for ecological specialization in *C. gattii*. Extracellular fibrils formation could be an adaptation for colonization on plant leaves, which offer an inhospitable surface for microbial colonization due to limited nutrients, extreme exposure to temperature changes, dehydration and radiation, and unique inter-specific competition [4], [60]. The inference for leaf colonization is supported by the fact that leaf colonization is a wide-spread trait in basidiomycetous yeasts especially many *Cryptococcus* species from the *Tremellales* lineage that includes *C. gattii* [61].

*C. gattii* infections are most likely acquired from host exposure to environments where the fungus grows on or in association with leaves and trees especially tree hollows [62]. We sought to model this situation in the laboratory, by growing *C. gattii* on plant substrates, and then examining its virulence in a murine model of cryptococcosis. We utilized growth on *A. thaliana* leaf agar, which induced *C. gattii* to produce extracellular fibrils. Mice infected using C. gattii cells with extracellular fibrils had higher rates of lung and brain colonization by the fungus and significantly lower survival. Histopathological examination of diseased tissues also showed extensive fungal proliferation and destruction of pulmonary tissue in infected mice; the host response appeared rather limited, in terms of PMN infiltration. The ease with which we were able to produce a hypervirulent variant of the parent *C. gattii* strain, most notably for IN infection, by simply changing the growth substrate, underscored the crucial effect that natural habitat could exert as a modulator of innate virulence of a pathogen. Alternately, *C. gattii* grown on YPD agar did not express all putative virulence factors. Further experiments are warranted to clarify the roles that growth substrates exert on the virulence of *C. gattii*.

To examine cause-effect relationship between extracellular fibrils formation and hypervirulence by a complementary approach, we utilized in vitro fungus–PMN assay since this assay is rapid and results correlate well with virulence properties seen in animal model [31], [63]. A significant reduction in PMN -mediated killing of *C. gattii* cells grown on plant-based substrates showed expected correlation with the increased virulence seen in mice. Thus, two independent experimental approaches indicated that *C. gattii* with extracellular fibrils display hypervirulence trait. An insight into possible mechanism was achieved when fungus–neutrophils preparations were examined by SEM. We discovered extensive NETs in the presence of *C. gattii* cells with extracellular fibrils. This observation was significant given that NETs have not been described previously in any interactions with either *C. neoformans* or *C. gattii*. More surprisingly, the extent of NET formation did not correlate with fungal killing: more traps were seen in preparations with extracellular fibrils-bearing *C. gattii* cells that were less susceptible to neutrophils killing. This observation was surprising in view of previous publications on the pathogenic yeast *Candida albicans*, and several bacterial pathogens; NETs were found to be efficient weapons in PMN-mediated microbial killing [34], [64], [65]. Our experiments suggest that *C. gattii* extracellular fibrils associated virulence is the result of either inhibition of, or escape from, PMN-mediated killing [66]. Equally importantly, we have provided evidence that profuse NET formation does not always suffice to eliminate *C. gattii*. Our findings on neutrophils impairment are significant in view of early landmark studies by Murphy and colleagues [67], who reported an important distinction between *C. gattii* and *C. neoformans*; the former, but not the latter, could inhibit PMN chemotaxis and chemokinesis. Our studies also support a role for the microbial capsule in inhibition of NET-mediated killing, as shown earlier for bacteria; encapsulated *Streptococcus pneumoniae* can escape from the microbicidal action of NETs and from confinement in the lungs, and then cause systemic disease [68].

Additional studies are needed to expand our understanding of *C. gattii*–plant interactions, and relevance of such interactions to fungal virulence. For example, we know little about the nature of the host plant responses to the presence of the fungus; are plant defense mechanisms critical in permitting colonization? It would also be important to examine *C. gattii* mutant strains deficient in actin, tubulin and extra-cellular transport for characterization of extracellular fibrils formation. Immune effector cells other than neutrophils are also likely critical in allowing eventual survival and dissemination of *C. gattii* cells from lungs; do *C. gattii* extracellular fibrils interact with these cell types? In summary, we believe that examination of pathogenic fungi in conditions that simulate their specialized niches would be a fruitful approach to discern what distinguishes these pathogens from myriad of non-pathogenic fungi from similar environment. Increased knowledge of factors that modulate fungal virulence traits will eventually allow better design of antifungal drugs and vaccines.

## Supporting Information

Figure S1 *C. gattii* colonized *A. thaliana* mutant plant leaves. *A. thaliana* ecotype Columbia (Col-0) and various mutant ecotypes such as *eds1* (enhanced disease susceptibility 1; lipase/signal transducer/triacylglycerol lipase), *nahG* (transgenic line degrading salicylic acid; SA), *npr1* (nonexpressor of PR genes 1; pathogenesis-related 1), *sid2* (SA-induction deficient), *rpm1* (resistance to *Pseudomonas syringae pv maculicola* 1), and *pad4* (phytoalexin deficient 4) were grown in a greenhouse at the Biology Department, Syracuse University, Syracuse, NY (1–3, 5). Four-to six-week old plants were transferred to the Mycology Laboratory of the Wadsworth Center where they were maintained at 20–23°C with a 12 hr light/dark cycle under 50–70% humidity, in a modified incubator with HEPA filtration. *C. gattii* cells were subcultured twice in YPD broth at 30°C with 180 rpm shaking and were then collected by centrifugation, washed twice in deionized sterile water (DSW) and re-suspended to a concentration of 1.0×10^6^ cells/mL. Four to six leaves on each *A. thaliana* plant were lightly wounded on the adaxial surface on either side of the mid-vein with a 27-gauge syringe needle (4, 6, 7). Two 5-µl drops of 10^6^ *C. gattii* cells/mL were placed at the wound site, and allowed to air dry (5–10 min). Plants were replaced in modified growth incubator maintained at 20–23°C, 12 hr light/dark cycle, and 50–70% humidity. After 7 days, inoculated plants were transferred to a BSL 2 cabinet, leaves excised and photographed with a digital camera. Whole plants and close up of inoculated leaves showed varying levels of scars. (References [1.Cao, H., S. A. Bowling, A. S. Gordon, and X. Dong. 1994. Characterization of an *Arabidopsis* mutant that is nonresponsive to inducers of systemic acquired resistance. Plant Cell 6:1583–1592]; [2.Delaney, T. P., S. Uknes, B. Vernooij, L. Friedrich, K. Weymann, D. Negrotto, T. Gaffney, M. Gut-Rella, H. Kessmann, E. Ward, and J. Ryals. 1994. A central role of salicylic acid in plant disease resistance. Science 266:1247–1250]; [3.Falk, A., B. J. Feys, L. N. Frost, J. D. Jones, M. J. Daniels, and J. E. Parker. 1999. *EDS1*, an essential component of R gene-mediated disease resistance in *Arabidopsis* has homology to eukaryotic lipases. Proc Natl Acad Sci U S A 96:3292-7]; [4.Fullner, K. J. 1998. Role of *Agrobacterium virB* genes in transfer of T complexes and RSF1010. J Bacteriol 180:430-4]; [5.Grant, M. R., L. Godiard, E. Straube, T. Ashfield, J. Lewald, A. Sattler, R. W. Innes, and J. L. Dangl. 1995. Structure of the *Arabidopsis RPM1* gene enabling dual specificity disease resistance. Science 269:843-6]; [6. Mithofer, A., G. Wanner, and W. Boland. 2005. Effects of Feeding *Spodoptera littoralis* on lima bean leaves. II. Continuous mechanical wounding resembling insect feeding is sufficient to elicit herbivory-related volatile emission. Plant Physiol. 137:1160–1168]; [7.Polizzi, G., and A. Vitale. 2006. *Dasylirion serratifolium* as a new host of *Botrytis cinerea*, the causal agent of leaf spots and blight in Italy. Plant Disease 90:114–114]).
(8.56 MB DOC)

Figure S2 *C. gattii* showed enhanced colonization of *A. thaliana* mutant plant leaves. A number of mutant plants with genotypes derived from *A. thaliana* Col-0 ecotype were inoculated as described in supplementary figure S1. Inoculated leaves were homogenized in glass tissue grinders, homogenate suspended in sterile deionized water and a series of dilutions plated on YPD agar for recovery of fungal colony forming units (CFU). The mutant plants showed higher susceptibility to colonization with *C. gattii* wild-type cells. The colonization was highest in *eds1* and *nahG* mutants. Overall, colonization was *eds1*>*nahG*>*sid2*>*npr1*>*rpm1*. The experiment was repeated once.
(0.14 MB DOC)

Figure S3 *C. gattii* extracellular projections were formed on agar with plant substrates. Dialysis tubing was cut to small squares approximately 1 cm×1 cm and sterilized by boiling in water for 20–30 min. Three squares of sterilized tubing were then laid flat over YPD, *A. thaliana* leaf, black cherry wood chip or Niger seed agar plates. Each square was inoculated with 100 µl of 10^7^ cells/ml *C. gattii* wild-type cells. Plates were allowed to dry and incubated at 25°C for 4 days. Small blocks of agar (∼1 cm×1 cm) were removed and fixed in 2% glutaraldehyde 0.2 M sodium cacodylate buffer, and dehydrated by graded alcohol series, critical point dried, gold sputter coated and imaged by. (A) YPD agar, (B) *A. thaliana* leaf agar, (C) Niger seed agar, and (D) Black cherry wood chip agar. SEM micrographs of one representative *C. gattii* cell imaged from (E) YPD agar, (F) *A. thaliana* leaf agar, (G) Niger seed agar, and (H) Black cherry wood chip agar, scale bar 1 µm. Notably, *C. gattii* extracellular fibrils were absent on YPD agar in contrast to abundant formation on agar supplemented with plant substrates.
(3.15 MB DOC)

Figure S4 *C. gattii* extracellular fibrils are altered by cytoskeletal protein inhibitors. We examined the roles of actin, tubulins and other cytoskeletal proteins on the formation of *C. gattii* extracellular fibrils since these proteins are important determinants of cell shape (1, 3). *C. gattii* wild-type and *cap59Δ* mutant strain were grown in YPD broth for 12–16 hr. Thiabendazole (25–150 µg/ml), cytochalasin B (25–150 µg/ml), mebandazol (20–80 µg/ml), or latrunculin B (100 µ/ml–400 µM/ml), were added to individual cultures and incubated for an additional 6 hr (2). An aliquot of yeast cells were also treated with 2 M sodium azide at 65°C for 30 min to render them non-viable. Cells were collected by centrifugation, washed twice with SDW, and re-suspended to a concentration of 10^8^ cells/ml. Cells were serially diluted in SDW and plated on YPD agar to determine any loss in viability. None of the drug treatments caused significant loss of viability. Six to eight leaves each from at least three different *A. thaliana* plants were scratch wounded and inoculated with two 5-µl drops of 1×10^6^ *C. gattii* cells obtained from each treatment described above. Controls included leaves from wounded plants inoculated with SDW, and non-wounded, non-inoculated *A. thaliana* plants. Leaves were harvested 7-days post inoculation and prepared for light microscopy and SEM as described in the previous section. Microfilament (Actin) and microtubule (Tubulin) inhibitors cytochalasin B (Top left), latrunculin B (Top right), thiabendazole (Bottom left) or mebandazole (Bottom right) alter *C. gattii* cell attachment and extracellular fibril formation in a concentration dependent manner. Lower magnification (left panel) and higher magnification images (right panel) for each treatment are shown with red borders demarcating original inoculation sites. The concentrations used were cytochalasin B (Top left panels [A–B 25 µg/mL], [C–D 50 µg/mL], [E–F 100 µg/mL], and [G–H 150 µg/mL]); latrunculin B (Top right panels [A–B 100 µM], [C–D 200 µM] and [E–F 400 µM]); thiabendazole (Bottom left panels [A–B 25 µg/mL], [C–D 50 µg/mL], [E–F 100 µg/mL], and [G–H 150 µg/mL]); mebandazole (Bottom right panels [A–B 20 µg/mL], [C–D 40 µg/mL], [E–F 60 µg/mL], and [G–H 80 µg/mL]). A concentration dependent effect on extracellular fibril formation is most pronounced with latrunculin and mebendazole. (References[1.Gao, L., and A. Bretscher. 2009. Polarized growth in budding yeast in the absence of a localized formin. Mol. Biol. Cell 20:2540–2548];[2.Muchowski, P. J., K. Ning, C. D'Souza-Schorey, and S. Fields. 2002. Requirement of an intact microtubule cytoskeleton for aggregation and inclusion body formation by a mutant Huntingtin fragment. Proc Natl Acad Sci U S A 99:727–32];[3.Nelson, W. J. 2003. Adaptation of core mechanisms to generate cell polarity. Nature 422:766–774].
(8.02 MB DOC)

Figure S5 Cytoskeleton protein inhibitor drugs disrupt extracellular fibrils. We tested for the disruptive effects of the cytoskeleton-inhibitory drugs on *C. gattii* cells, utilizing the Thermonox® plastic cover slip assay. Thiabendazole (25–150 µg/ml), cytochalasin B (25–150 µg/ml), mebandazol (20–80 µg/ml), or latrunculin B (100 µM/ml–400 µM/ml), were added to individual cultures and incubated for an additional 6 hr (details in figure S4). Treated cells were washed and re-suspended to a final concentration of 1×10^6^ cell/ml in minimum asparagine broth supplemented with 1% glucose, and incubated in 24-well plates. Thermanox® plastic coverslips were then processed for SEM. Left column displays view of a typical cell and right column displays lower magnification micrograph of a group of cells and the morphology of microtubes. Cytochalasin B 25 µg/mL, latrunculin B 100 µM, mebandazole 10 µg/mL, and thiabendazole 25 µg/mL; scale equals 1.0 µm. Disorganization and inhibition of extracellular fibril formation is evident with all drug treatments.
(3.39 MB DOC)

Figure S6 Enhanced colonization of brain and lung tissues by *C. gattii* cells grown on leaf agar. BALB/c mice were inoculated either via IV (A, B) or IN (C, D) route with either 30 µl or 100 µl containing 10^4^ (IN) or 10^5^ (IV) *C. gattii* cells passage two times on YPD, Niger seed (NGS), *A. thaliana* leaf (LEAF), or black cherry wood chip agar (WOOD). Mice were sacrificed 7 days (IV) or 14 days (IN) post infections and CFUs per gram brain or lung tissue was determined. Notably, enhanced colonization of brain and lung tissues by *C. gattii* grown on *A. thaliana* leaf agar is independent of the route of infection.
(0.21 MB DOC)
