# Supplementary figures and images for: Extracellular Fibrils of Pathogenic Yeast Cryptococcus gattii Are Important for Ecological Niche, Murine Virulence and Human Neutrophil Interactions
Source: PLoS One. 2010 Jun 7;5(6):e10978. doi: 10.1371/journal.pone.0010978 (PMC2881863; doi:10.1371/journal.pone.0010978)

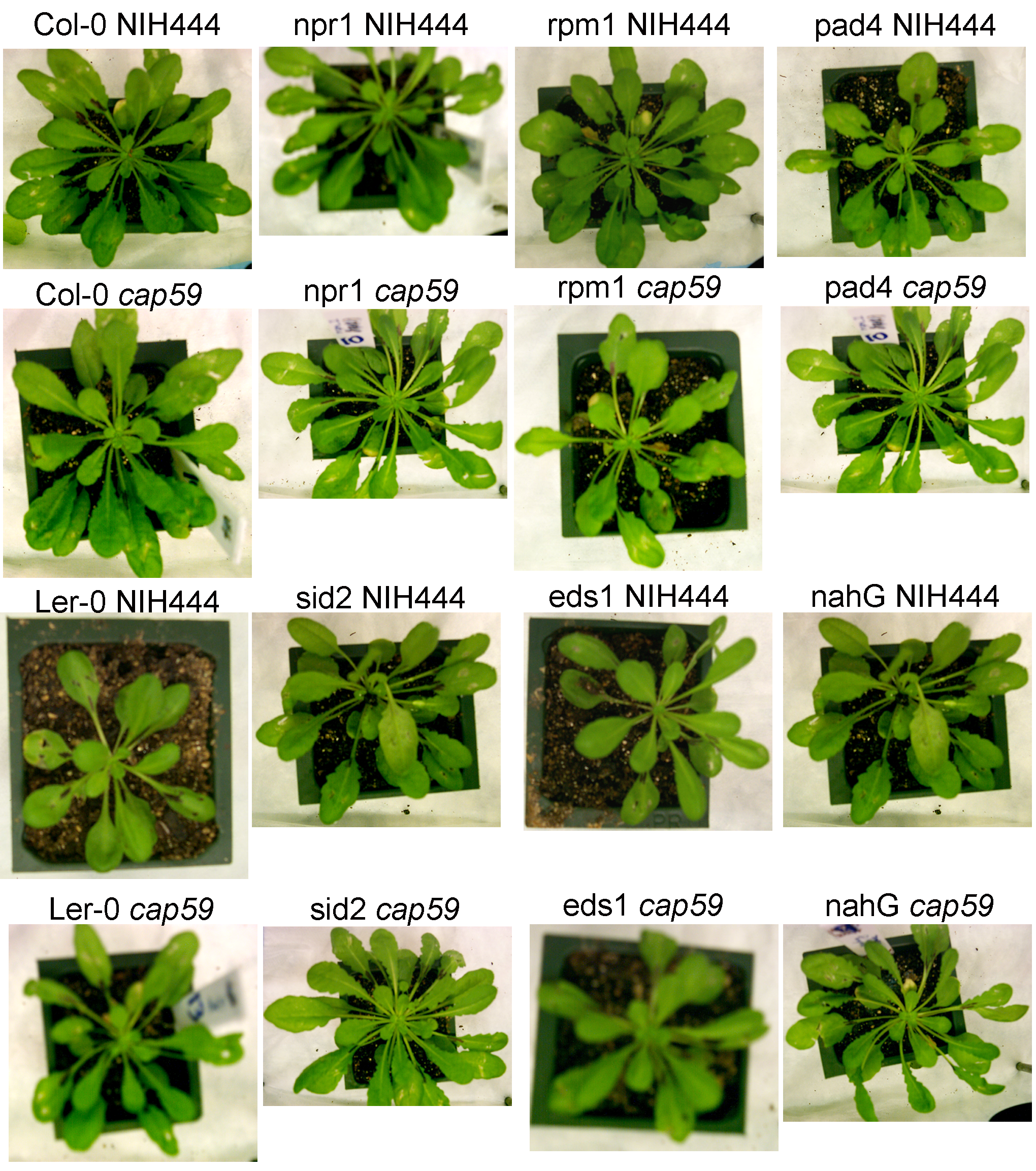

Supplement: Figure S1 — C. gattii colonized A. thaliana mutant plant leaves. A. thaliana ecotype Columbia (Col-0) and various mutant ecotypes such as eds1 (enhanced disease susceptibility 1; lipase/signal transducer/triacylglycerol lipase), nahG (transgenic line degrading salicylic acid; SA), npr1 (nonexpressor of PR genes 1; pathogenesis-related 1), sid2 (SA-induction deficient), rpm1 (resistance to Pseudomonas syringae pv maculicola 1), and pad4 (phytoalexin deficient 4) were grown in a greenhouse at the Biology Department, Syracuse University, Syracuse, NY (1–3, 5). Four-to six-week old plants were transferred to the Mycology Laboratory of the Wadsworth Center where they were maintained at 20–23°C with a 12 hr light/dark cycle under 50–70% humidity, in a modified incubator with HEPA filtration. C. gattii cells were subcultured twice in YPD broth at 30°C with 180 rpm shaking and were then collected by centrifugation, washed twice in deionized sterile water (DSW) and re-suspended to a concentration of 1.0×10^6^ cells/mL. Four to six leaves on each A. thaliana plant were lightly wounded on the adaxial surface on either side of the mid-vein with a 27-gauge syringe needle (4, 6, 7). Two 5-µl drops of 106 C. gattii cells/mL were placed at the wound site, and allowed to air dry (5–10 min). Plants were replaced in modified growth incubator maintained at 20–23°C, 12 hr light/dark cycle, and 50–70% humidity. After 7 days, inoculated plants were transferred to a BSL 2 cabinet, leaves excised and photographed with a digital camera. Whole plants and close up of inoculated leaves showed varying levels of scars. (References [1.Cao, H., S. A. Bowling, A. S. Gordon, and X. Dong. 1994. Characterization of an Arabidopsis mutant that is nonresponsive to inducers of systemic acquired resistance. Plant Cell 6:1583–1592]; [2.Delaney, T. P., S. Uknes, B. Vernooij, L. Friedrich, K. Weymann, D. Negrotto, T. Gaffney, M. Gut-Rella, H. Kessmann, E. Ward, and J. Ryals. 1994. A central role of salicylic acid in plant d [file pone.0010978.s001.doc]

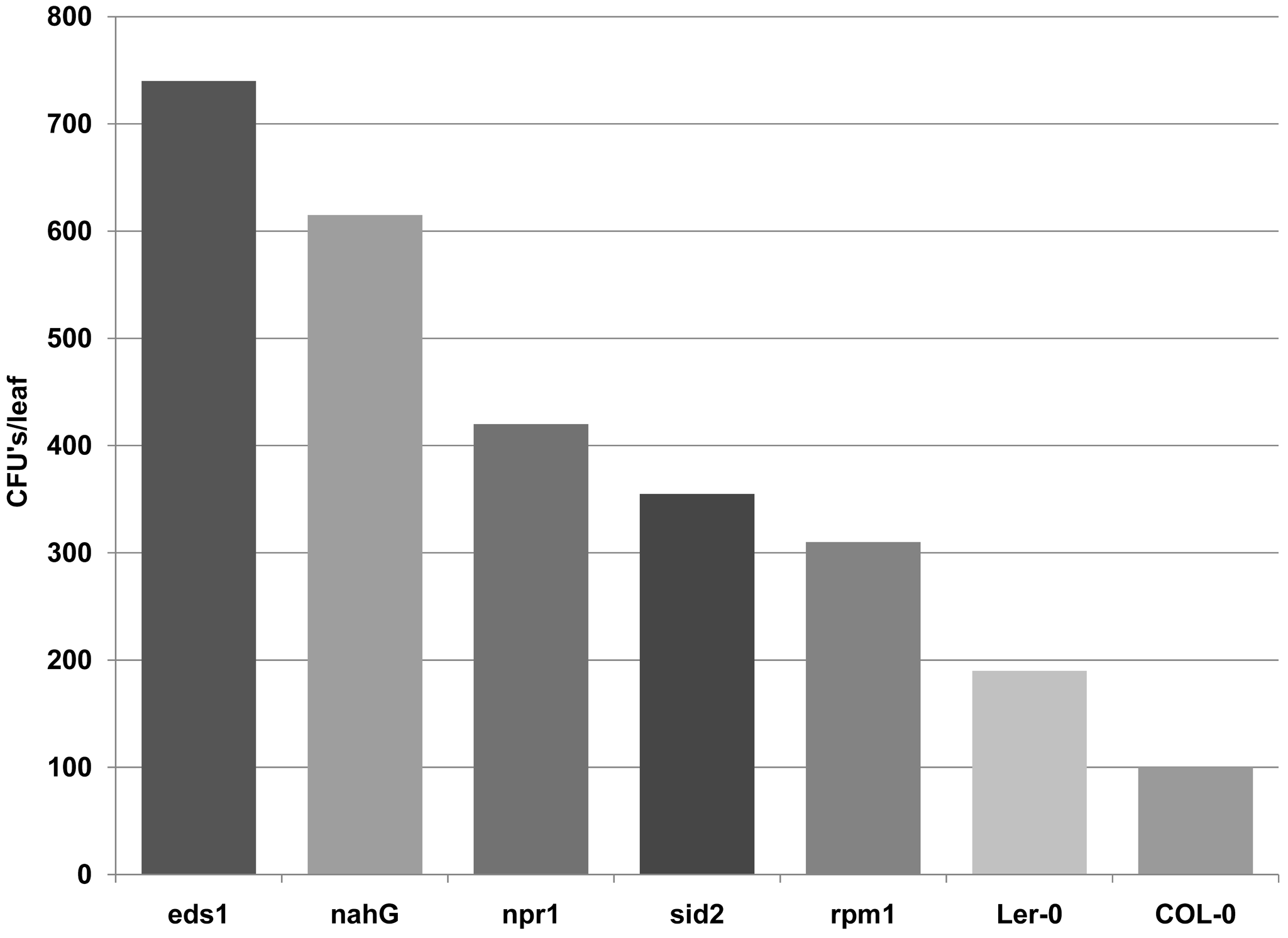

Supplement: Figure S2 — C. gattii showed enhanced colonization of A. thaliana mutant plant leaves. A number of mutant plants with genotypes derived from A. thaliana Col-0 ecotype were inoculated as described in supplementary figure S1. Inoculated leaves were homogenized in glass tissue grinders, homogenate suspended in sterile deionized water and a series of dilutions plated on YPD agar for recovery of fungal colony forming units (CFU). The mutant plants showed higher susceptibility to colonization with C. gattii wild-type cells. The colonization was highest in eds1 and nahG mutants. Overall, colonization was eds1>nahG>sid2>npr1>rpm1. The experiment was repeated once. (0.14 MB DOC) [file pone.0010978.s002.doc]

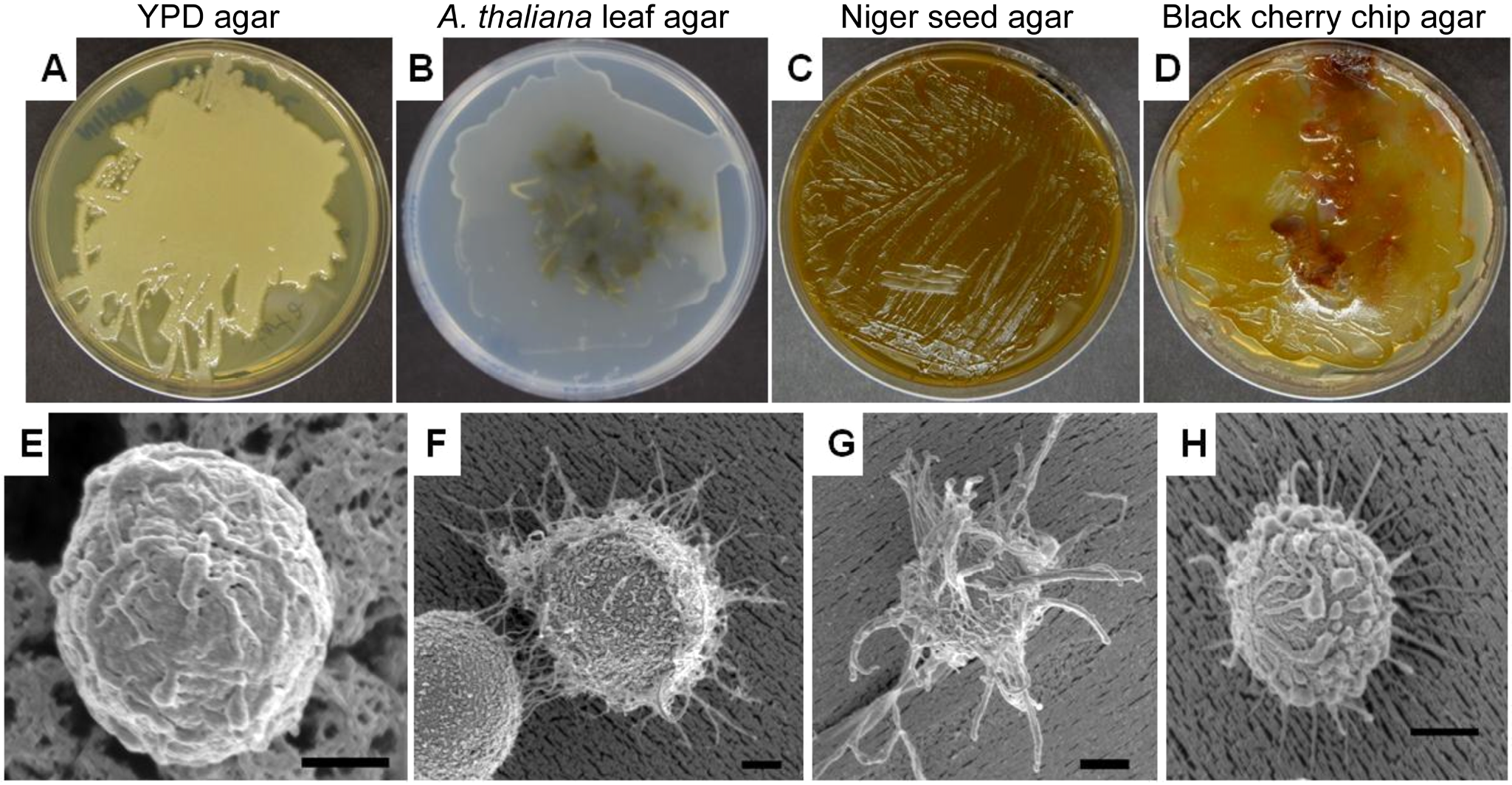

Supplement: Figure S3 — C. gattii extracellular projections were formed on agar with plant substrates. Dialysis tubing was cut to small squares approximately 1 cm×1 cm and sterilized by boiling in water for 20–30 min. Three squares of sterilized tubing were then laid flat over YPD, A. thaliana leaf, black cherry wood chip or Niger seed agar plates. Each square was inoculated with 100 µl of 107 cells/ml C. gattii wild-type cells. Plates were allowed to dry and incubated at 25°C for 4 days. Small blocks of agar (∼1 cm×1 cm) were removed and fixed in 2% glutaraldehyde 0.2 M sodium cacodylate buffer, and dehydrated by graded alcohol series, critical point dried, gold sputter coated and imaged by. (A) YPD agar, (B) A. thaliana leaf agar, (C) Niger seed agar, and (D) Black cherry wood chip agar. SEM micrographs of one representative C. gattii cell imaged from (E) YPD agar, (F) A. thaliana leaf agar, (G) Niger seed agar, and (H) Black cherry wood chip agar, scale bar 1 µm. Notably, C. gattii extracellular fibrils were absent on YPD agar in contrast to abundant formation on agar supplemented with plant substrates. (3.15 MB DOC) [file pone.0010978.s003.doc]

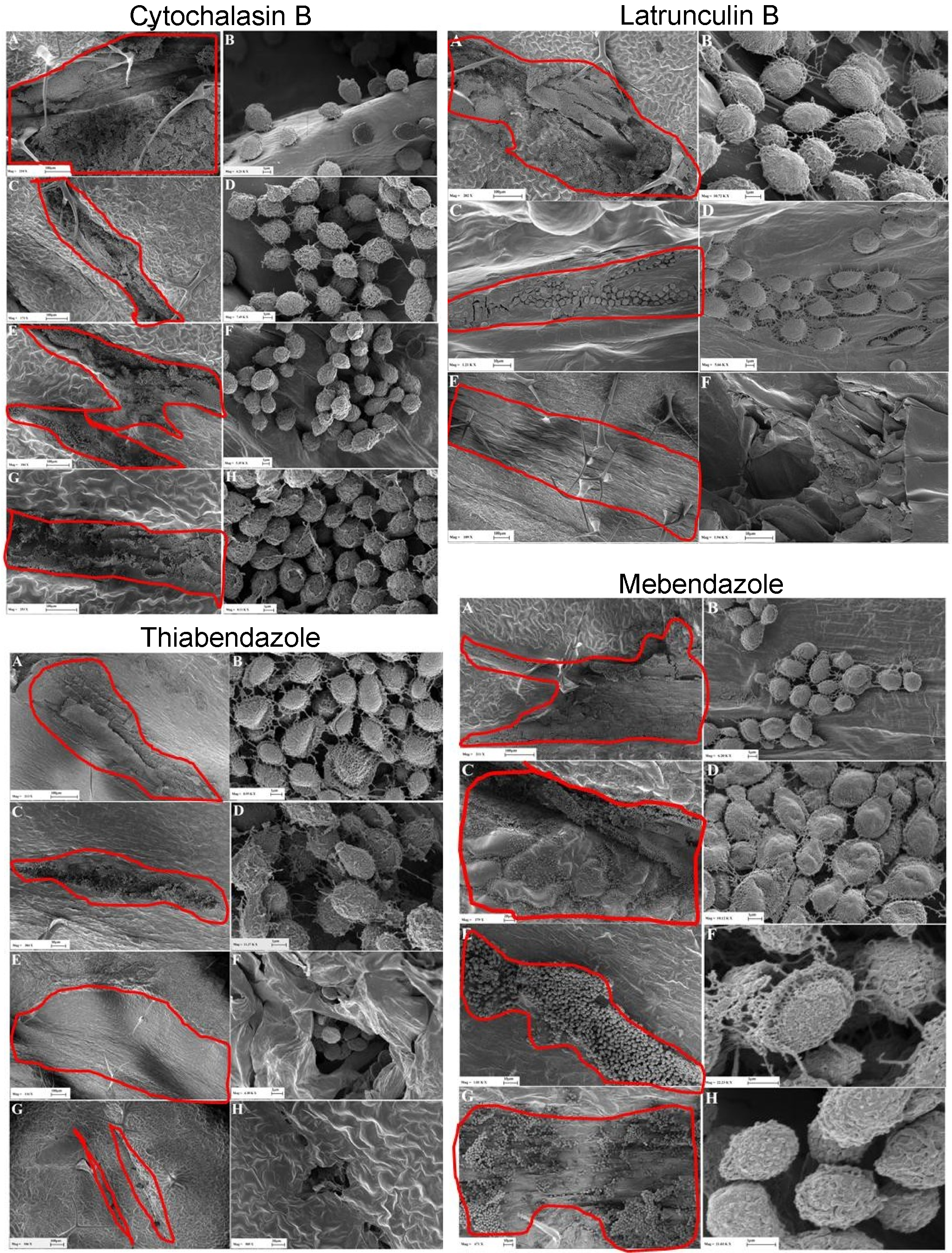

Supplement: Figure S4 — C. gattii extracellular fibrils are altered by cytoskeletal protein inhibitors. We examined the roles of actin, tubulins and other cytoskeletal proteins on the formation of C. gattii extracellular fibrils since these proteins are important determinants of cell shape (1, 3). C. gattii wild-type and cap59Δ mutant strain were grown in YPD broth for 12–16 hr. Thiabendazole (25–150 µg/ml), cytochalasin B (25–150 µg/ml), mebandazol (20–80 µg/ml), or latrunculin B (100 µ/ml–400 µM/ml), were added to individual cultures and incubated for an additional 6 hr (2). An aliquot of yeast cells were also treated with 2 M sodium azide at 65°C for 30 min to render them non-viable. Cells were collected by centrifugation, washed twice with SDW, and re-suspended to a concentration of 108 cells/ml. Cells were serially diluted in SDW and plated on YPD agar to determine any loss in viability. None of the drug treatments caused significant loss of viability. Six to eight leaves each from at least three different A. thaliana plants were scratch wounded and inoculated with two 5-µl drops of 1×10^6^ C. gattii cells obtained from each treatment described above. Controls included leaves from wounded plants inoculated with SDW, and non-wounded, non-inoculated A. thaliana plants. Leaves were harvested 7-days post inoculation and prepared for light microscopy and SEM as described in the previous section. Microfilament (Actin) and microtubule (Tubulin) inhibitors cytochalasin B (Top left), latrunculin B (Top right), thiabendazole (Bottom left) or mebandazole (Bottom right) alter C. gattii cell attachment and extracellular fibril formation in a concentration dependent manner. Lower magnification (left panel) and higher magnification images (right panel) for each treatment are shown with red borders demarcating original inoculation sites. The concentrations used were cytochalasin B (Top left panels [A–B 25 µg/mL], [C–D 50 µg/mL], [E–F 100 µg/mL], and [G–H 150 µg/mL]); latrunculin B (Top right panels [A– [file pone.0010978.s004.doc]

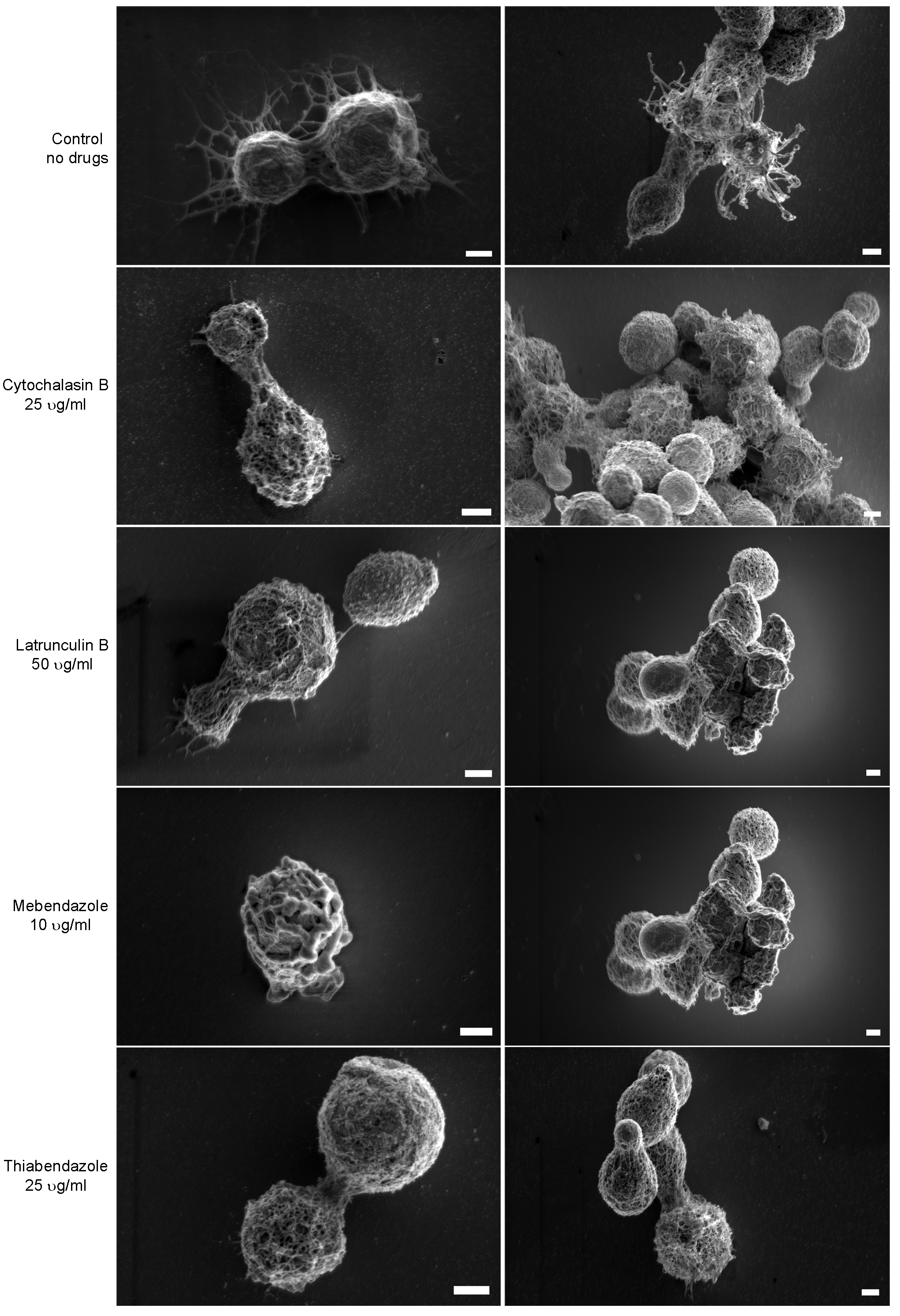

Supplement: Figure S5 — Cytoskeleton protein inhibitor drugs disrupt extracellular fibrils. We tested for the disruptive effects of the cytoskeleton-inhibitory drugs on C. gattii cells, utilizing the Thermonox® plastic cover slip assay. Thiabendazole (25–150 µg/ml), cytochalasin B (25–150 µg/ml), mebandazol (20–80 µg/ml), or latrunculin B (100 µM/ml–400 µM/ml), were added to individual cultures and incubated for an additional 6 hr (details in figure S4). Treated cells were washed and re-suspended to a final concentration of 1×10^6^ cell/ml in minimum asparagine broth supplemented with 1% glucose, and incubated in 24-well plates. Thermanox® plastic coverslips were then processed for SEM. Left column displays view of a typical cell and right column displays lower magnification micrograph of a group of cells and the morphology of microtubes. Cytochalasin B 25 µg/mL, latrunculin B 100 µM, mebandazole 10 µg/mL, and thiabendazole 25 µg/mL; scale equals 1.0 µm. Disorganization and inhibition of extracellular fibril formation is evident with all drug treatments. (3.39 MB DOC) [file pone.0010978.s005.doc]

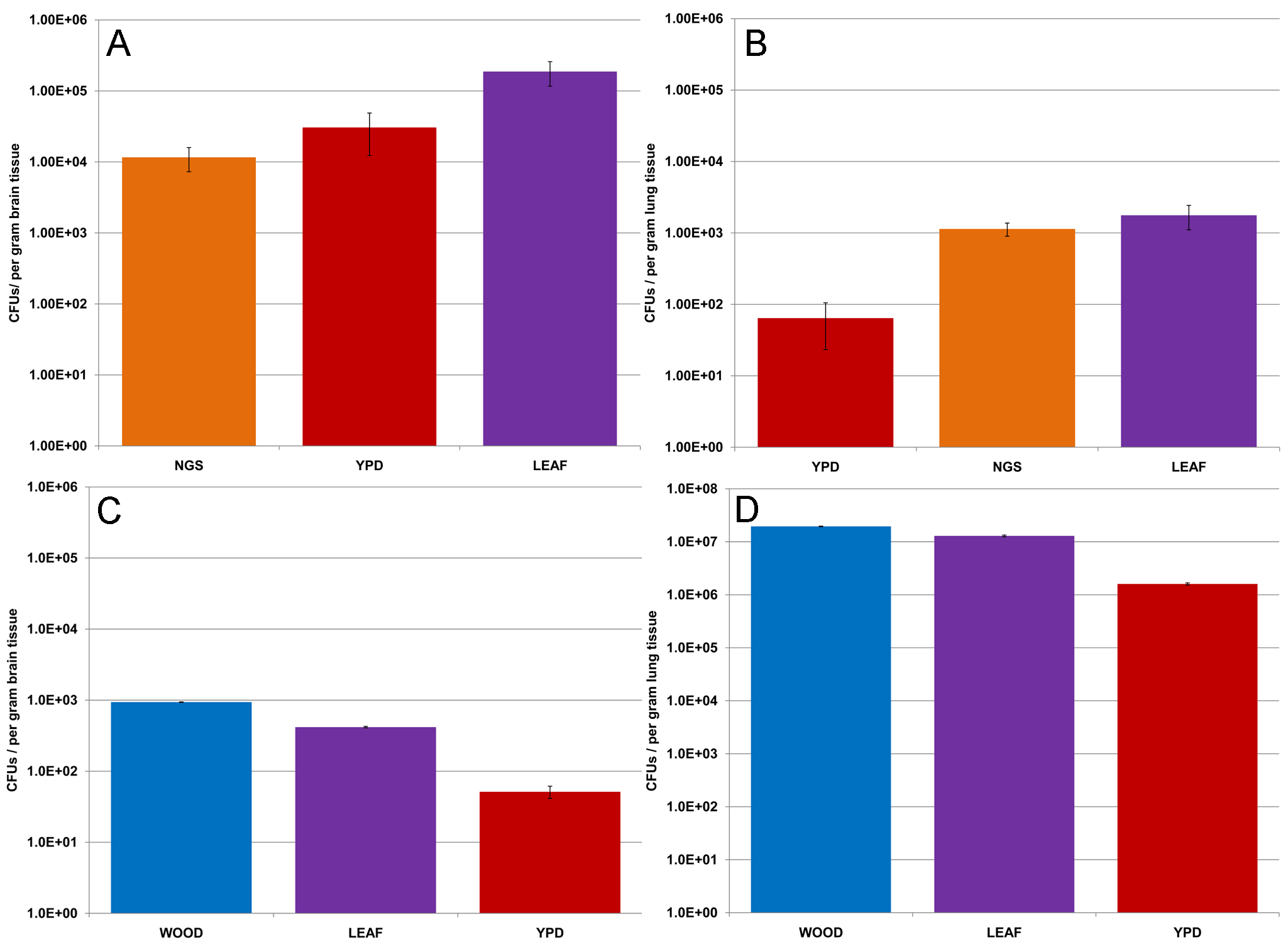


**Brain Tissue - IV**

**Lung Tissue - IV**

**Brain Tissue - IN**

**Lung Tissue - IN**

Supplement: Figure S6 — Enhanced colonization of brain and lung tissues by C. gattii cells grown on leaf agar. BALB/c mice were inoculated either via IV (A, B) or IN (C, D) route with either 30 µl or 100 µl containing 104 (IN) or 105 (IV) C. gattii cells passage two times on YPD, Niger seed (NGS), A. thaliana leaf (LEAF), or black cherry wood chip agar (WOOD). Mice were sacrificed 7 days (IV) or 14 days (IN) post infections and CFUs per gram brain or lung tissue was determined. Notably, enhanced colonization of brain and lung tissues by C. gattii grown on A. thaliana leaf agar is independent of the route of infection. (0.21 MB DOC) [file pone.0010978.s006.doc]
